# Perception of castration value over cost in the metastatic prostate cancer scenario: a contemporary pharmacoeconomic perspective

**DOI:** 10.1590/S1677-5538.IBJU.2021.0258

**Published:** 2021-06-15

**Authors:** Luciana Saboya Brito Dal Col, Danilo L. Andrade, Lucas M. Gon, Diego M. Capibaribe, Marcelo P. Amaro, Natássia C. C. Truzzi, Barbara R. Malkomes, Leonardo O. Reis

**Affiliations:** 1 Universidade Estadual de Campinas Campinas SP Brasil UroScience, Universidade Estadual de Campinas – UNICAMP, Campinas, SP, Brasil; 2 Pontifícia Universidade Católica de Campinas Departamento de Urologia Campinas SP Brasil Departamento de Urologia, Pontifícia Universidade Católica de Campinas - PUC - Campinas, Campinas, SP, Brasil

## INTRODUCTION

Prostate cancer is the most prevalent cancer in the male population, and although it may have a mainly indolent course, it can also progress to metastatic cancer, which has a median survival of 42 months (1-3).

The first line of treatment for metastatic prostate cancer aims to eliminate testosterone’s stimulatory effect (4) and inhibit disease progression. Both surgical and pharmacological approaches can achieve testosterone castration levels. With appropriate treatment, men with prostate cancer can live longer, making it necessary to minimize the risks of harm, considering general health, sexuality, psychological, and economic effects in the long term (5,6).

Financial toxicity is an exponentially growing concern as it directly influences the quality of life and treatment adherence, culminating with wasted resources, and suboptimal clinical outcomes.

However, about one-third of the oncologists express a high degree of discomfort discussing costs with patients (7-11) and both caregivers and patients avoid discussing cost-related issues due to factors such as potential bias to treatment recommendations. In metastatic prostate cancer disease, urologists play an essential role in helping patients decide the best treatment. It is crucial to consider the treatment’s economic impact, beyond clinical factors, patient desires, and expectations.

To further understand the underuse of surgical castration (12) even among urologists, we consecutively and anonymously interviewed one hundred urologists during a urology meeting. They answered a survey with multiple-choice questions regarding their perceptions of value, cost, and pharmacoeconomic issues concerning metastatic prostate cancer castration treatment.

## MATERIALS AND METHODS

Briefly, a questionnaire assessed the environment of practice (public, private, or academic) and the country region. The urologists were asked whether they treated patients with metastatic prostate cancer, and the current percentage for chemical or surgical castration use, totalizing 100%. Also, if the remuneration for the surgical castration were ten times higher than it is, how would this percentage change, and the three main reasons for choosing medical castration. The next question asked: by opting for surgical castration, how many avoided pharmacological castrations could save enough resources for one patient to have access to a second-generation antiandrogen.

ANOVA compared repeated measures by means across one or more variables, and the Cohen test measured the effect size using the SAS System for Windows (Statistical Analysis System), 9.4 version, SAS Institute Inc, Cary, NC, USA.

## RESULTS

Among one hundred urologists screened for the study, 34 either did not treat prostate cancer routinely or refrained from participating. Among the participants, 37.8% had more than 15 years of practice, and 21.2% worked in an academic center.

Pharmacological castration is regularly offered for 78.05% of patients (mean, SD 28.06, median 90%); 62.5% in academic versus 82.2% in non-academic centers, p=018. That rate would drop significantly to 54.21% (mean, SD 38.88, median 50.00%, p <0.0001), a major effect (0.88) on Cohen’s test, if money compensation for the procedure would increase 10-fold.

Those that considered surgical castration cost-effective (60.6%), are significantly more susceptible to financial incentives (p=0.036). In contrast, those who usually choose pharmacological castration due to convenience, remuneration, and modernity are considerably less susceptible (p=0.044).

When asked about the number of surgical castrations needed to save enough money to provide one patient with access to a second-generation antiandrogen the answers were: the rationale is not valid by n=23 (34.85%), 5-15 by n=29 (43.93%), and 16-25 by n=14 (21.21%) urologists.

## DISCUSSION

Financial toxicity can be as devastating as other adverse medical events, causing patient distress, morbidity and mortality. Surgical castration can be significantly less expensive than pharmacological with similar oncological and functional impact (13).

However, chemical castration is still the preferred choice, even for patients who need lifelong androgen deprivation therapy (14). The current study shows that it is regularly offered for 78.05% of patients (mean, SD 28.06, median 90%); 62.5% in academic versus 82.2% in non-academic centers, p=018.

Urologists infrequently offered patients a choice or considered patient’s beliefs and preferences, implying limited patient participation in the management of their care as previously reported (15). By not offering choice, the opportunity for a shared decision making is hampered. On the other hand, the percentage of pharmacological castration offered would significantly drop if surgical castration was best remunerated. This could be one reason why many urologists still choose pharmacological treatment over orchiectomy, considering the significant influence of pharmaceutical industries in this specific field, while surgical treatment is still inadequately compensated.

Surgical and pharmacological castration has shown similar oncological results. Still, men are supposedly more prone to refuse orchiectomy based on the cosmetics and psychological factors regarding self-image and its irreversibility.

Though controversial, according to Sun et al. (13), pharmacological castration was associated with significantly higher risks of fractures, peripheral arterial disease, diabetes mellitus, and cardiac-related complications when compared to surgery. Bonzani et al. (16) have found no difference when comparing the quality of life and body image among medically and surgically castrated patients. Potosky et al. (17) showed that men were more likely to worry about their disease and overall health with treatment, reinforcing the importance of considering long-term implications including costs while choosing between surgical or pharmacological treatment.

Orchiectomy is highly effective, outpatient surgery, low cost, low morbidity, and the treatment is warranted essentially in case of drug shortage, financial issues, or patient non-adherence to the treatment. One must also consider that surgery can achieve castration levels faster than medication, and it is not vulnerable to patient adherence or medication availability. Nonetheless, it still suffers massive critic from the interested parties. It is easy to realize that a simple tactic such as improving the medical compensation for surgical castration and also including a testicular prosthesis to the “orchiectomy kit” would convert it to a significant overall economy and patient benefit (18,19).

The costs and resources of public and private health care are discrepant. In the public system in Brazil, a subcapsular orchiectomy outpatient surgery costs the government (at a lifetime) on average USD 83.00 (R$ 453.62) while chemical castration per year costs USD 950.00 (R$ 5.200.00), not considering treatment of eventual surgical complications or medication side effects (20). In many places, the government does not provide the medicine being this cost passed on to the patient or generating legal processes that further increase spending.

**Figure 1:**
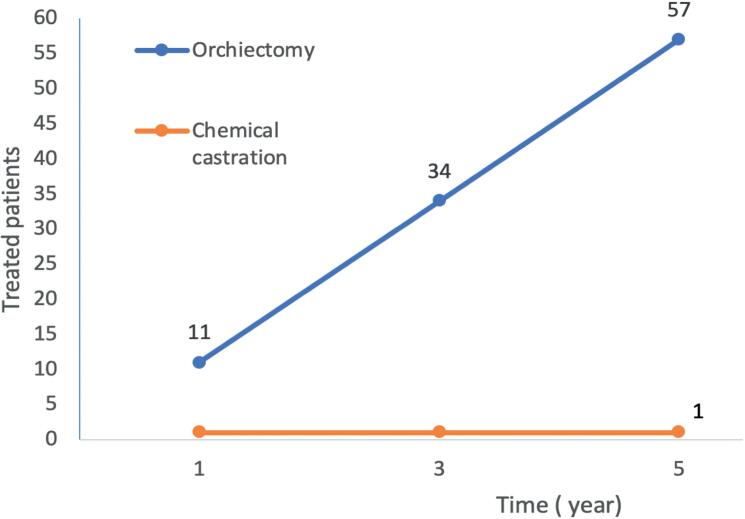
Number of patients treated with same investment comparing surgical and pharmacological castration (20).

One patient at chemical castration spends money enough to treat about 11 patients with surgical castration in a year and 57 patients in 5 years (Figure-1). From a different perspective, offering surgical castration to 30 patients (in opposition to chemical) will save money enough to treat at least 1 castration-resistant patient with second-generation antiandrogen for a lifetime. While numbers might vary, the cost-effective trend for surgical castration is time sensible and exponential.

Though the potential reversibility is considered a central argument favoring the pharmacological castration, it is theoretical once the hypothalamic-testicle axis takes months or years to awake from chronic inhibition and testosterone replacement warrants an immediate, effective, cheap and safe recover in body composition, lipid parameters, and quality of life (21,22).

Another subject of interest is the potential effect of drug interactions considering that many patients have comorbidities and face polypharmacy (23). In the metastatic non-curable disease scenario, the surgical castration irreversibility is not an issue, and it can save money for sequential treatments once financial burden impacts not only the individual but also the overall healthcare system.

As a random consecutive interview during a national congress, our study reflects a sample from the Brazilian urology community. It may not represent the whole community view, but it can point out some directions for further studies and awareness actions, like cost-effectiveness in uro-oncology and strategies to encourage treatment modifications.

It is remarkable the coercion power of the medical device and pharmaceutical industry in medical behaviors. Improve medical remuneration can provide the best oncological outcome for the patient and the community. Moreover, most of the doctors have limited pharmacoeconomic knowledge, which is necessary to support the decisions and discussion with patients.

Pharmacological castration is still the preferred therapy among urologists when it comes to metastatic prostate cancer, despite having similar oncological results, higher costs, and potential for more collateral effects than orchiectomy. The described study results showed that the urology community is sensible to the fact that cost is not necessarily translated to value in this scenario. The pharmacoeconomic awareness is fundamental and should enlighten discussions concerning metastatic prostate cancer treatment in both public and private sectors, in addition to adapting the reality of medical conduct in a developing country that struggles to provide free health care to the entire population.

An unbiased patient centered decision is vital in the contemporary treatment landscape of castration resistant prostate cancer and additional strategies to improve patient care and fight financial toxicity beyond the surgical castration value perception might involve bipolar androgen therapy with potential to enhance quality of life, prolong disease stabilisation, postpone and improve the magnitude and duration of response to second-generation antiandrogens to maximise therapeutic benefit to patients (24).
